# Supplementary material for: Disordered proteins interact with the chemical environment to tune their protective function during drying
Source: bioRxiv. 2024 Mar 1:2024.02.28.582506. Preprint. [Version 1] doi: 10.1101/2024.02.28.582506 (PMC10925285; doi:10.1101/2024.02.28.582506)
Supplement: Supplementary file 1 [file NIHPP2024.02.28.582506v1-supplement-1.pdf]

bioRxiv preprint doi: https://doi.org/10.1101/2024.02.28.582506; this version posted March 2, 2024. The copyright holder for this preprint (which was not certified by peer review) is the author/funder, who has granted bioRxiv a license to display the preprint in perpetuity. It is made available under aCC-BY 4.0 International license.

## Supplemental Figures

bioRxiv preprint doi: https://doi.org/10.1101/2024.02.28.582506; this version posted March 2, 2024. The copyright holder for this preprint (which was not certified by peer review) is the author/funder, who has granted bioRxiv a license to display the preprint in perpetuity. It is made available under aCC-BY 4.0 International license.

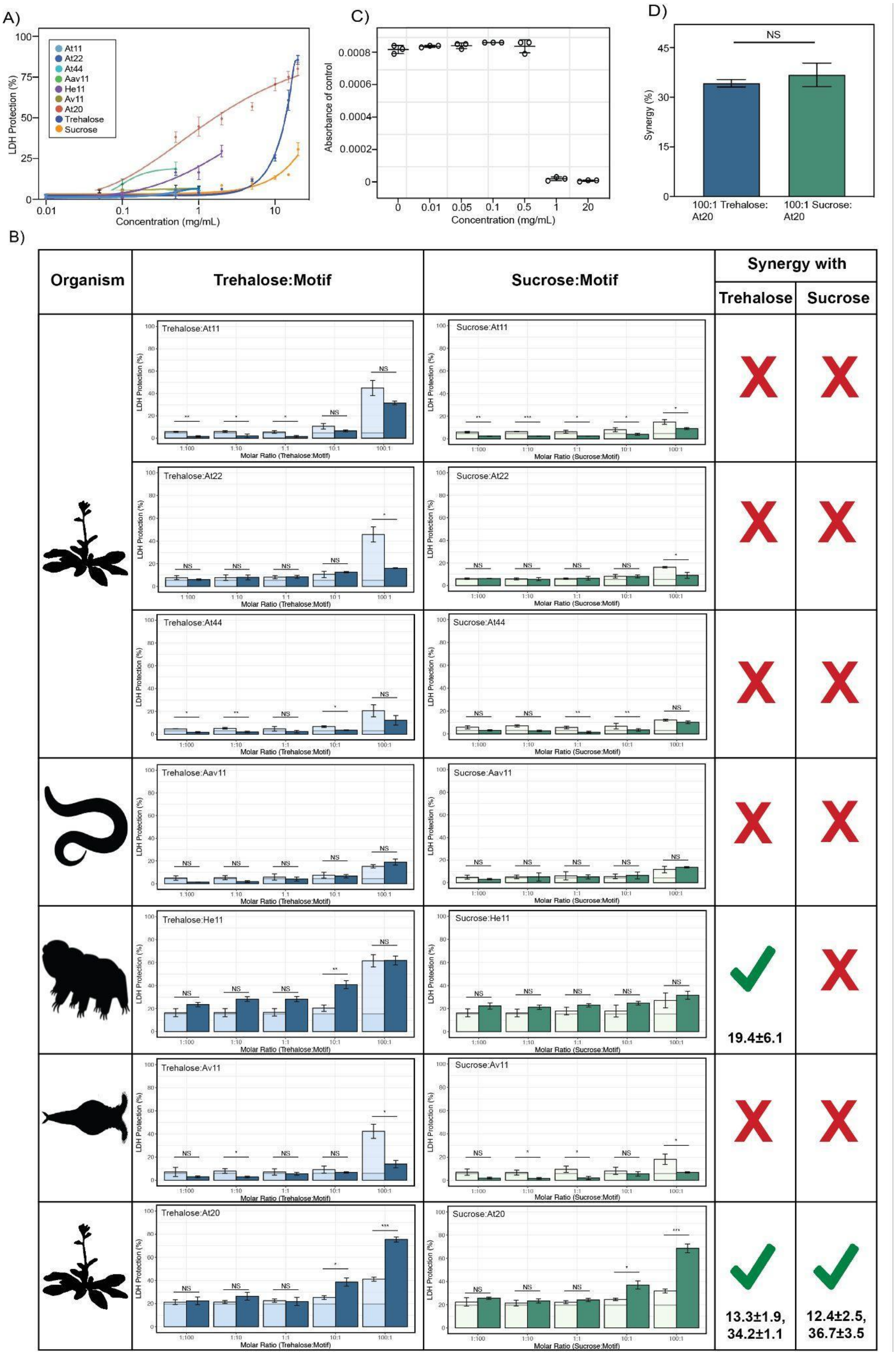

bioRxiv preprint doi: https://doi.org/10.1101/2024.02.28.582506; this version posted March 2, 2024. The copyright holder for this preprint (which was not certified by peer review) is the author/funder, who has granted bioRxiv a license to display the preprint in perpetuity. It is made available under aCC-BY 4.0 International license.

**Figure S1. A)** Sigmoidal plot representing concentration dependence of LDH protection by LEA motifs and cosolutes as a function of the weight/volume concentration **B)** Synergy plots for cosolutes with LEA motifs at 1:100, 1:10, 1:1, 10:1, and 100:1 molar ratios. n=3, Welch's t-test was used for statistical comparison, error bars = standard deviation. Numbers under the checkmarks represent the mean percentage synergy and standard deviation **C)** Change in NADH absorbance values for the refrigerated controls of Aav11 at different concentrations after 16 hours **D)** Percent synergy comparison for At20 with trehalose and sucrose at 100:1 ratio. Welch's t-test was used for statistical comparison.

bioRxiv preprint doi: https://doi.org/10.1101/2024.02.28.582506; this version posted March 2, 2024. The copyright holder for this preprint (which was not certified by peer review) is the author/funder, who has granted bioRxiv a license to display the preprint in perpetuity. It is made available under aCC-BY 4.0 International license.

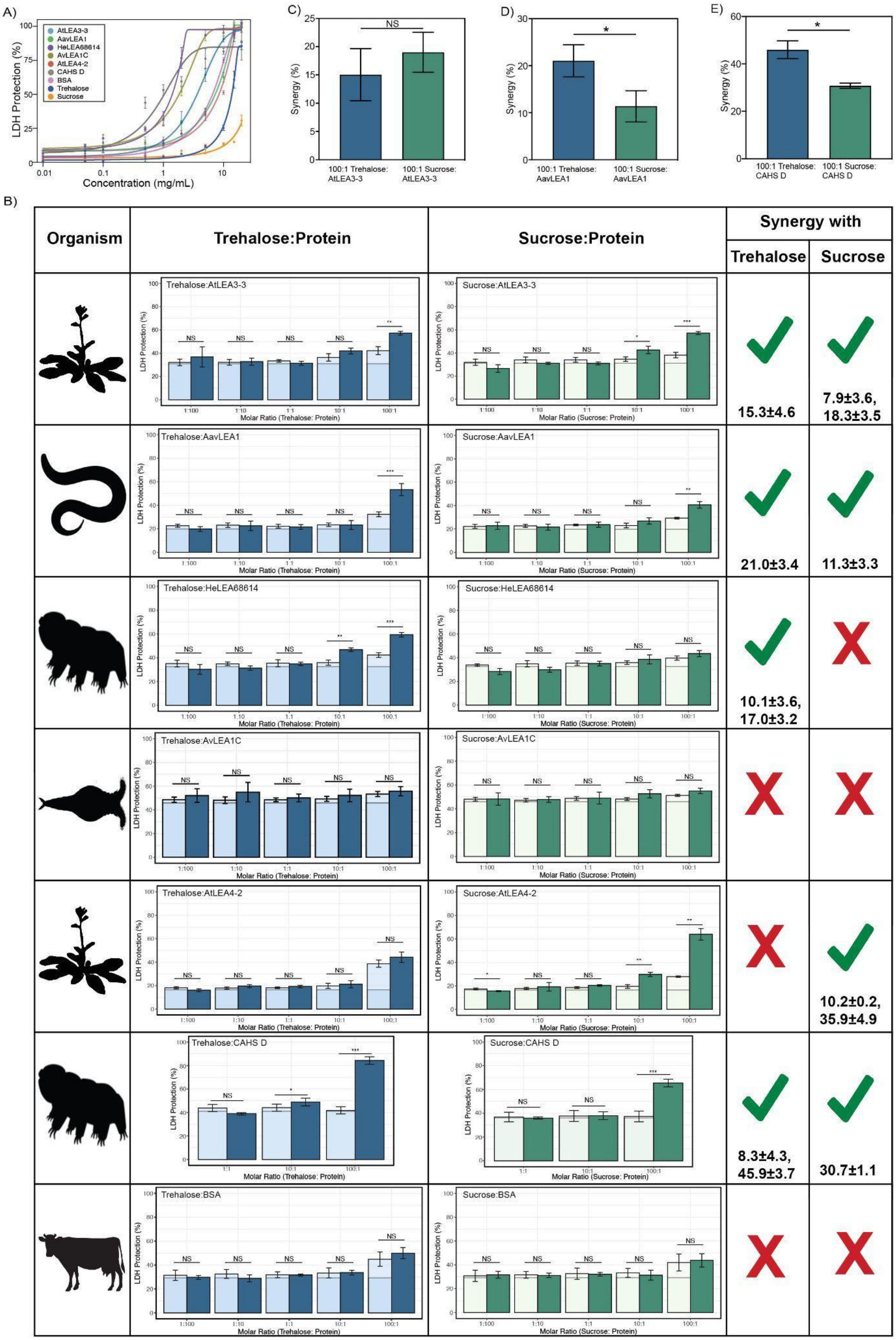

bioRxiv preprint doi: https://doi.org/10.1101/2024.02.28.582506; this version posted March 2, 2024. The copyright holder for this preprint (which was not certified by peer review) is the author/funder, who has granted bioRxiv a license to display the preprint in perpetuity. It is made available under aCC-BY 4.0 International license.

**Figure S2. A)** Sigmoidal plot representing concentration dependence of LDH protection by full-length proteins and cosolutes as a function of the weight/volume concentration. **B)** Synergy plots for cosolute with full-length proteins at respective molar ratios. n=3, Welch's t-test was used for statistical comparison, error bars = standard deviation. Numbers under the checkmarks represent the mean percentage synergy and standard deviation **C-E)** Percent synergy comparison for **C)** AtLEA3-3 **D)** AavLEA1 **E)** CAHS D with trehalose and sucrose at 100:1 ratio. n=3, error bars= standard deviation. Welch's t-test was used for statistical comparison.

bioRxiv preprint doi: https://doi.org/10.1101/2024.02.28.582506; this version posted March 2, 2024. The copyright holder for this preprint (which was not certified by peer review) is the author/funder, who has granted bioRxiv a license to display the preprint in perpetuity. It is made available under aCC-BY 4.0 International license.

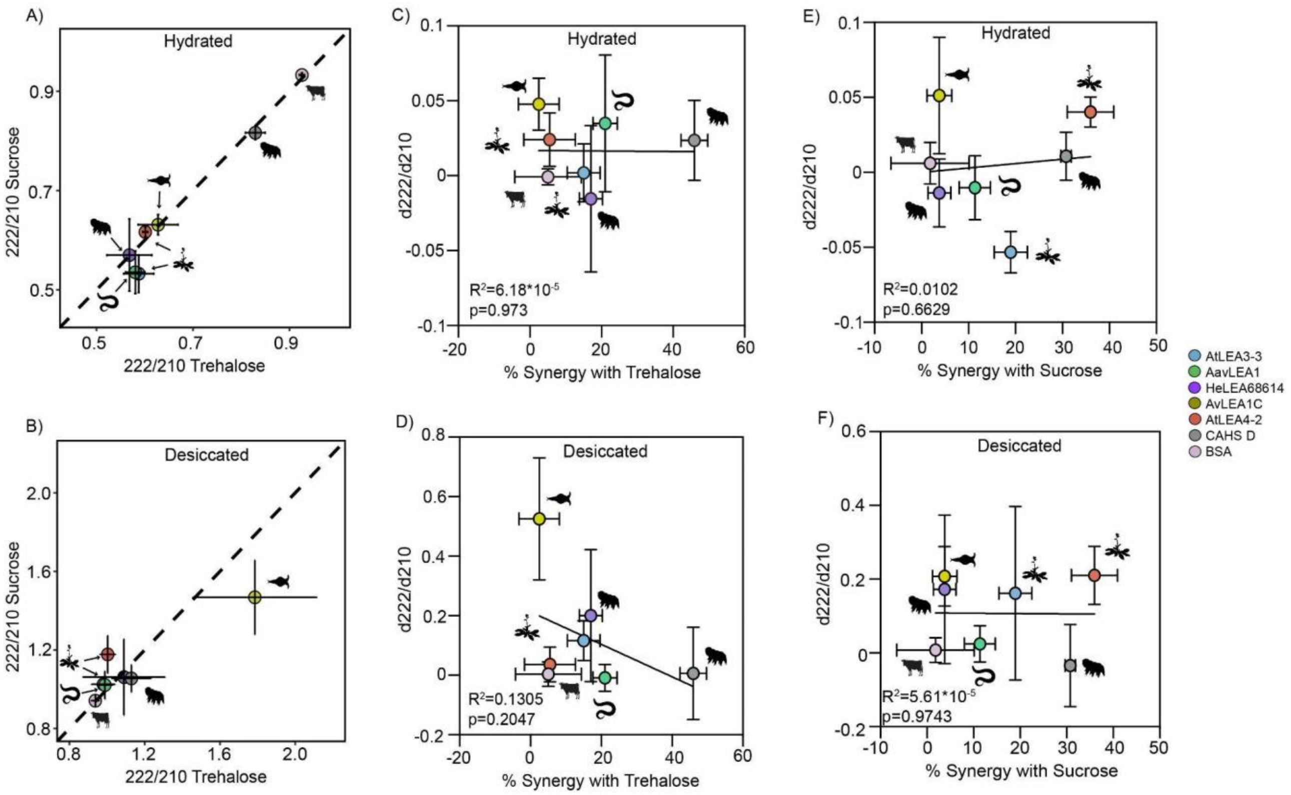


**Figure S3. A)** Plot comparing secondary structural changes in proteins with trehalose vs. sucrose at 100:1 molar ratio under hydrated conditions. Dotted line represents cases where there is equal structural shift in both trehalose and sucrose mixture. **B)** Plot comparing secondary structural alterations in proteins with trehalose vs. sucrose at 100:1 molar ratio under desiccated conditions. Dotted line represents cases where there is equal structural shift in both trehalose and sucrose mixture. **C)** Correlation plots for synergy vs. secondary structural shift in presence of trehalose in the hydrated state. **D)** Correlation plots for synergy vs. secondary structural shift in presence of trehalose in the desiccated state. **E)** Correlation plots for synergy vs. secondary structural shift in presence of sucrose in the hydrated state. **F)** Correlation plots for synergy vs. secondary structural shift in presence of sucrose in the desiccated state.

bioRxiv preprint doi: https://doi.org/10.1101/2024.02.28.582506; this version posted March 2, 2024. The copyright holder for this preprint (which was not certified by peer review) is the author/funder, who has granted bioRxiv a license to display the preprint in perpetuity. It is made available under aCC-BY 4.0 International license.

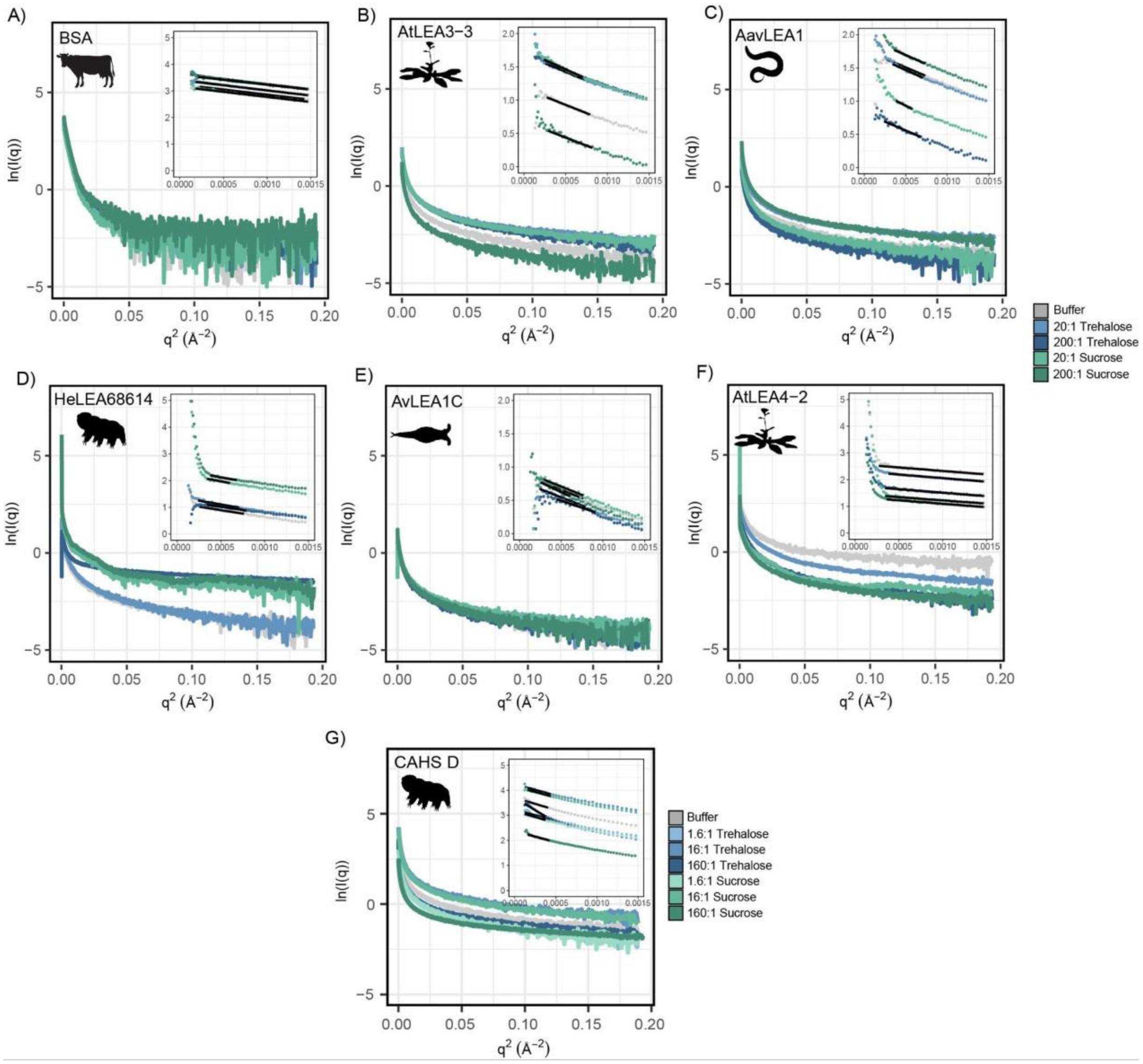


**Figure S4.** Raw SAXS data from the experiments shown in Fig. 4 for 4 mg/mL of **A)** BSA (0.0578 mM), **B)** AtLEA3-3 (0.22 mM), **C)** AavLEA1 (0.249 mM), **D)** HeLEA68614 (0.156 mM), **E)** AvLEA1C (0.163 mM), **F)** AtLEA4-2 (0.38 mM), and **G)** CAHS D (0.156 mM). Each plot displays raw SAXS data on a Guinier scale, with a zoomed-in portion in the top right displaying the Guinier region. The Guinier fit is displayed as a black trendline. In each plot, the protein's scattering profile in several solution environments is shown, including protein in the presence of no cosolute (black), a 20:1 molar ratio of trehalose (light blue), a 200:1 molar ratio of trehalose (dark blue), a 20:1 molar ratio of sucrose (light green), a 200:1 molar ratio of sucrose (dark green). CAHS D data contains slightly different molar ratios of 1.6:1 trehalose (very light blue), 16:1 trehalose (light blue), 160:1 trehalose (dark blue), 1.6:1 sucrose (very light green), 16:1 sucrose (light green), 160:1 sucrose (dark green). Unlike other samples, a Guinier analysis with a $qMaxR_g$ of 1.1 could not be obtained. A best attempt to establish the $R_g$ of this sample ($qMaxR_g$ ~ 1.35) revealed a significant increase in the $R_g$. Given the clear curvature of the Guinier region, this data was consistent with the presence of large oligomeric species.

bioRxiv preprint doi: https://doi.org/10.1101/2024.02.28.582506; this version posted March 2, 2024. The copyright holder for this preprint (which was not certified by peer review) is the author/funder, who has granted bioRxiv a license to display the preprint in perpetuity. It is made available under aCC-BY 4.0 International license.

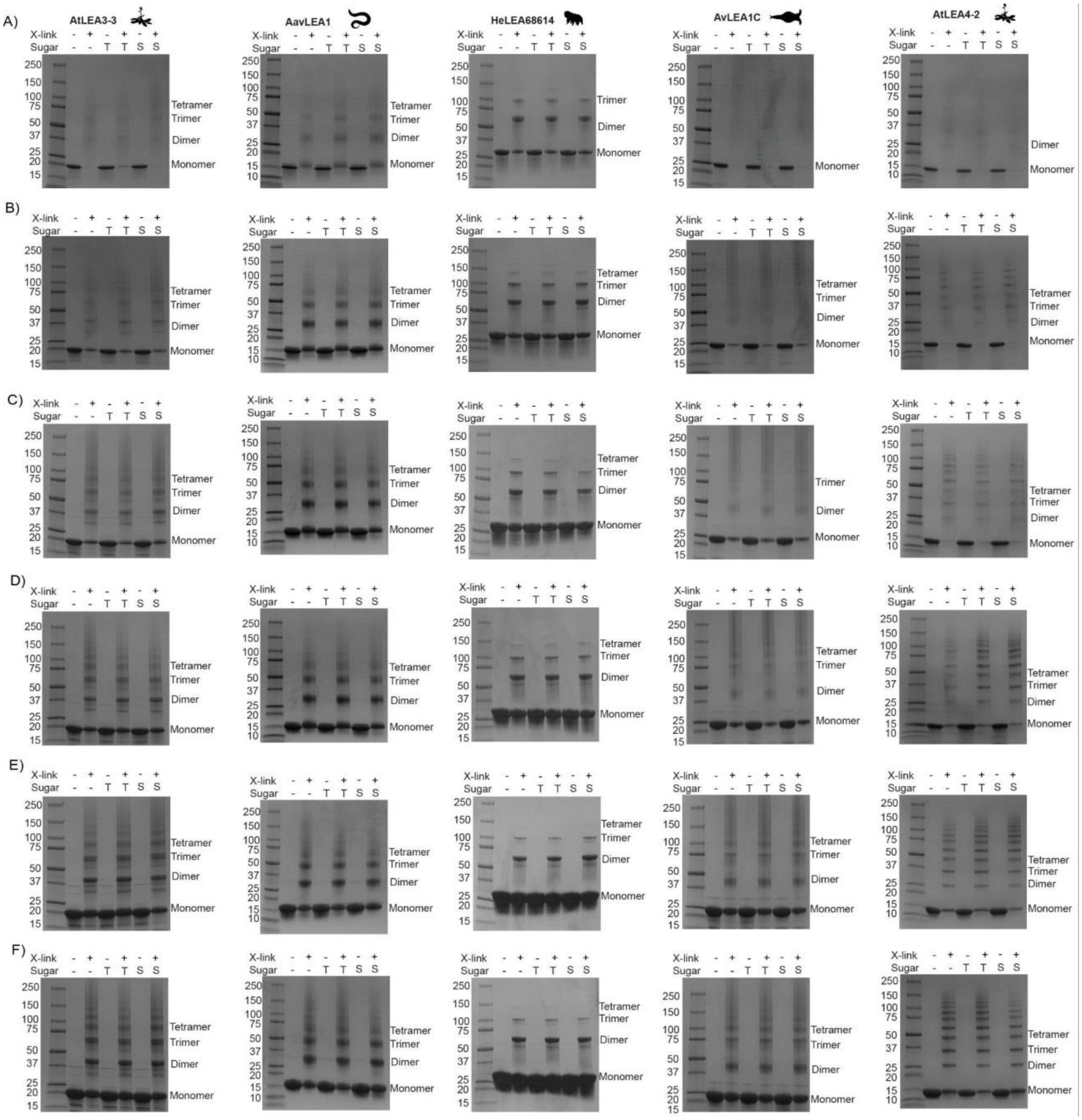


**Figure S5.** Crosslinking gels for full-length LEA proteins and in mixtures with trehalose or sucrose. A range of protein concentrations were used to cover the concentrations used in the synergy assay. Protein concentrations used were **A)** 25 μM, **B)** 50 μM, **C)** 75 μM, **D)** 100 μM, **E)** 150 μM, and **F)** 200 μM. Each gel contains protein ladder (Precision Plus Protein$^{TM}$ Dual Xtra Standards, Bio Rad, Catalog #161-0377) in the first lane, protein at desired concentration in the second lane, crosslinked protein in the third lane, 100:1 trehalose:protein in the fourth lane, crosslinked 100:1 trehalose:protein in the fifth lane, 100:1 sucrose:protein in the sixth lane, and crosslinked 100:1 sucrose:protein in the last lane.

bioRxiv preprint doi: https://doi.org/10.1101/2024.02.28.582506; this version posted March 2, 2024. The copyright holder for this preprint (which was not certified by peer review) is the author/funder, who has granted bioRxiv a license to display the preprint in perpetuity. It is made available under aCC-BY 4.0 International license.

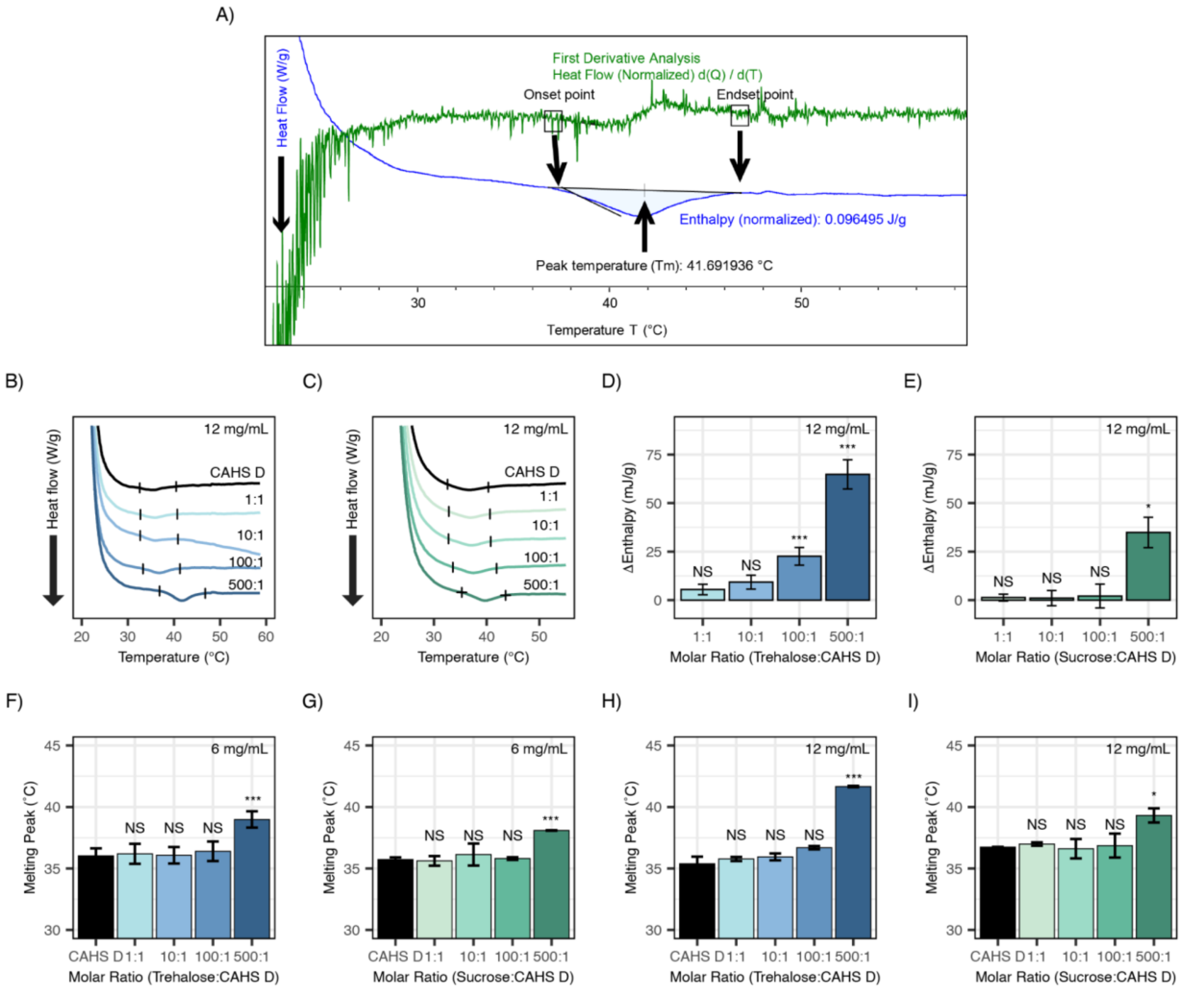


**Figure S6. A)** Sample enthalpy analysis using TRIOS software. First derivative analysis was used to aid in selecting the onset and endset in the thermogram representative of protein gelation. This is done by identifying observable spikes that correspond to where the curve begins to dip (onset) or level out (endset). The area between the approximate onset and endset is selected and TRIOS software is used to calculate defined onset and endset values, enthalpy (J/g), and peak temperature (Tm, ˚C) by analyzing the area above this feature within the identified range. **B)** DSC thermograms of CAHS D at 12 mg/mL (0.47 mM) in varying molar ratios of trehalose (0:1, 1:1, 10:1, 100:1, and 500:1). **C)** DSC thermograms of CAHS D at 12 mg/mL (0.47 mM) in varying molar ratios of sucrose (0:1, 1:1, 10:1, 100:1, and 500:1). **D)** Change in enthalpy measurements for trehalose:CAHS D mixtures relative to CAHS D at 12 mg/mL. Enthalpy measurements were done by taking the area of gel melt peaks represented by black dashes in Fig. S6B **E)** Change in enthalpy measurements for sucrose:CAHS D mixtures relative to CAHS D at 12 mg/mL. Enthalpy measurements were done by taking the area of gel melt peaks represented by black dashes in Fig. S6C. **(F-I)** Melting temperature for the gel melts as calculated from the DSC thermograms for CAHS D and cosolute:CAHS D mixtures at respective ratios.

bioRxiv preprint doi: https://doi.org/10.1101/2024.02.28.582506; this version posted March 2, 2024. The copyright holder for this preprint (which was not certified by peer review) is the author/funder, who has granted bioRxiv a license to display the preprint in perpetuity. It is made available under aCC-BY 4.0 International license.

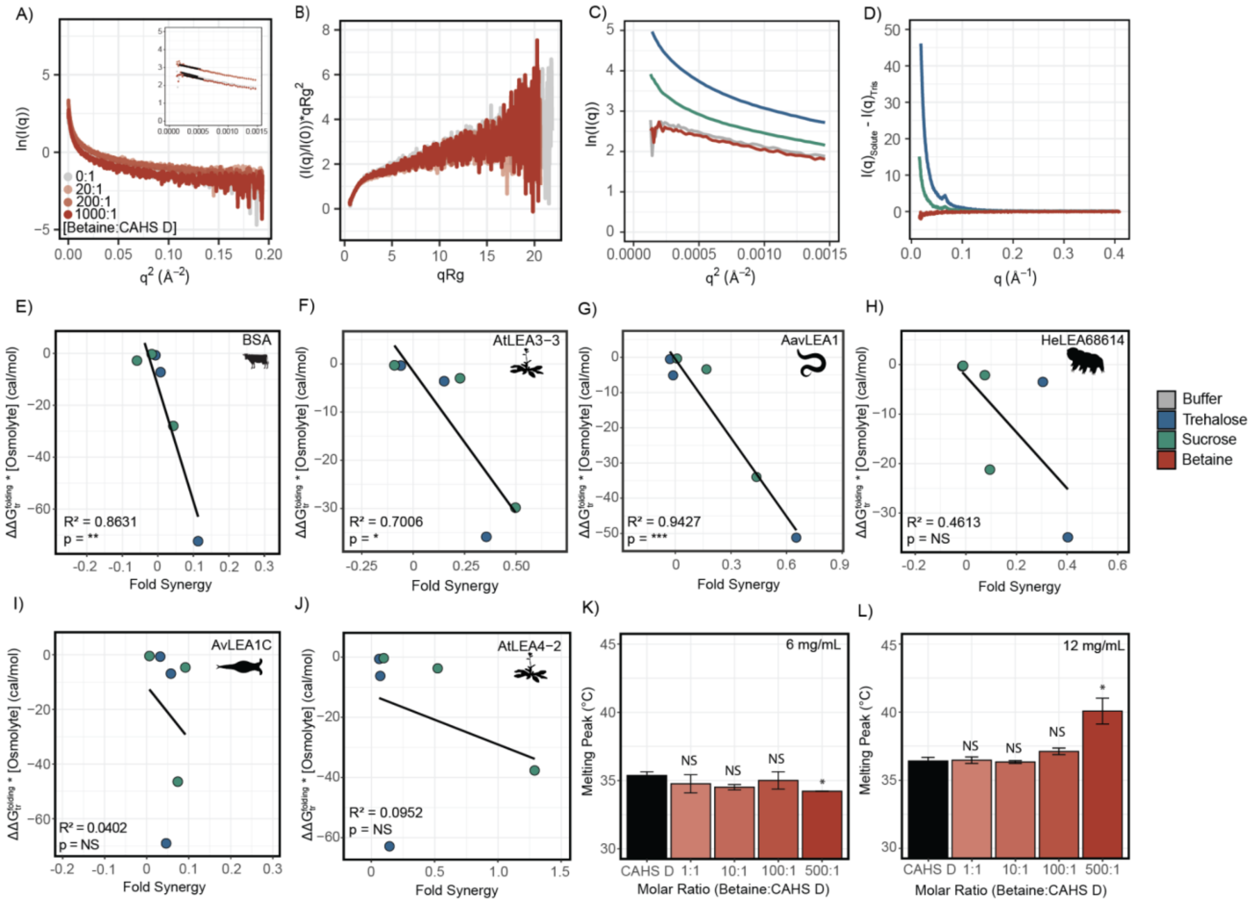


**Figure S7.** Raw SAXS data from the experiments shown in Fig. 6 for 4 mg/mL of CAHS D in various solution conditions. **A)** Scattering curves for CAHS D in tris (gray), 10:1 glycine betaine (light red), 100:1 glycine betaine (medium red), 1000:1 glycine betaine (dark red). Guinier regions are displayed in the top left with Guinier fits drawn in black. **B)** Dimensionless Kratky analysis of the data from A. **C)** Zoomed in Guinier regions of CAHS D in tris (gray), 1000:1 glycine betaine (red), 1000:1 trehalose (blue), and 1000:1 sucrose (green). **D)** Scattering profiles for CAHS D in glycine betaine, trehalose, and sucrose normalized to the protein's scattering profile in tris. **E-J)** Scatterplots of $\Delta\Delta G_{tr}^{folding}$ and synergy for BSA and LEA proteins. All fits are done using linear regression. All p-values derived from Pearson correlation. **K-L)** Melting temperature for the gel melts as calculated from the DSC thermograms for CAHS D and Betaine:CAHS D mixtures at respective ratios.

bioRxiv preprint doi: https://doi.org/10.1101/2024.02.28.582506; this version posted March 2, 2024. The copyright holder for this preprint (which was not certified by peer review) is the author/funder, who has granted bioRxiv a license to display the preprint in perpetuity. It is made available under aCC-BY 4.0 International license.

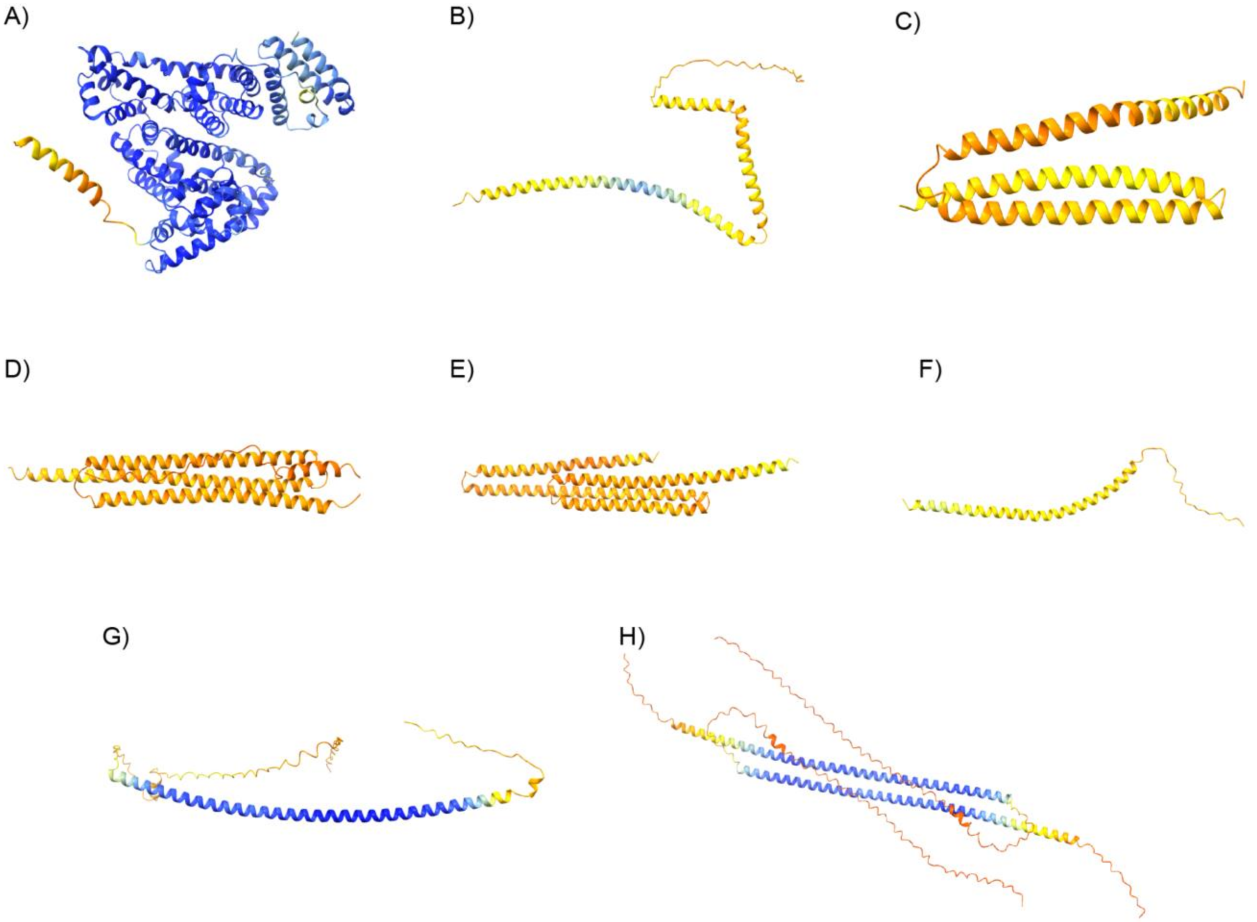


**Figure S8.** Representative images of the pdb structures obtained from our AlphaFold predictions. All structures are colored by their pLDDT on a scale from 0 (red) to 100 (blue). **A**) Monomeric BSA. **B**) Monomeric AtLEA3-3. **C**) Monomeric AavLEA1. **D**) Monomeric HeLEA68614. **E**) Monomeric AvLEA1C. **F**) Monomeric AtLEA4-2. **G**) Monomeric CAHS D. **H**) Dimeric CAHS D. See supplementary data for pdb files of each prediction & their exact pLDDT.

bioRxiv preprint doi: https://doi.org/10.1101/2024.02.28.582506; this version posted March 2, 2024. The copyright holder for this preprint (which was not certified by peer review) is the author/funder, who has granted bioRxiv a license to display the preprint in perpetuity. It is made available under aCC-BY 4.0 International license.

| Organism | Motif | Concentration (mM) |
|---|---|---|
| 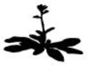 | At11 | 0.297 |
| | At22 | 0.277 |
| | At44 | 0.161 |
| 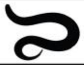 | Aav11 | 0.0562 |
| 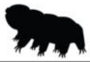 | He11 | 0.287 |
| 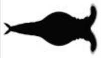 | Av11 | 0.262 |
| 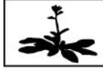 | At20 | 0.264 |

**Table S1.** Protein concentrations used in synergy assays for LEA motifs in this study.

| Organism | Protein | Concentration (mM) |
|---|---|---|
| 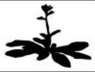 | AtLEA3-3 | 0.1215 |
| 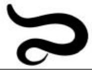 | AavLEA1 | 0.112 |
| 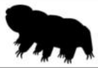 | HeLEA68614 | 0.0392 |
| 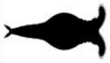 | AvLEA1C | 0.068 |
| 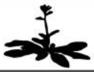 | AtLEA4-2 | 0.217 |
| 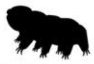 | CAHS D | 0.0078 |
| 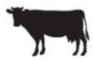 | BSA | 0.054 |

**Table S2.** Protein concentrations used in synergy assays for full-length proteins in this study.
